# Reflex Anoxic Seizures Induced by Needle Stick and Successfully Treated With Intranasal Midazolam

**DOI:** 10.7759/cureus.18002

**Published:** 2021-09-15

**Authors:** Lani Matthews, Mohan Kurukumbi

**Affiliations:** 1 Neurology, Virginia Commonwealth University School of Medicine, Richmond, USA; 2 Neurology, Inova Health System, Fairfax, USA

**Keywords:** convulsive syncope, asystole, seizure, venipuncture, midazolam, reflex asystolic syncope, vasovagal, reflex anoxic seizure, cardioinhibitory syncope

## Abstract

Reflex anoxic seizures are a type of convulsive syncope seen more commonly in children. In rare cases, they may develop into true epileptic seizures. There is no current consensus on treatment. In this case report, we present an adult patient with reflex anoxic seizures, along with EEG monitoring from an event. Our patient had a successful trial with intranasal midazolam preventing the episode, suggesting that anxiety played a significant role in her case. Reflex anoxic seizures in adults are rare and need to be properly identified, as treatment can lead to significant improvements in patient quality of life.

## Introduction

Reflex anoxic seizures (RAS), or reflex asystolic syncope, are a type of convulsive syncope triggered by an unpleasant stimulus. They are most often seen in children between six months and four years of age, with a prevalence of one in 1,000 [1]. About one-fourth of children with RAS continue to have episodes past their preschool years [2]. Clinically, after an unpleasant stimulus such as a bump to the head, the patient will become pallid often with cyanotic lips and lose consciousness. They usually have tonic posturing and irregular jerking movements, with the whole episode typically lasting less than a minute. The term “reflex anoxic seizure” is sometimes reserved for children, and in adults, an episode may be referred to as a convulsive cardioinhibitory neurally mediated syncope [2]. In some cases, the syncopal episode can induce a true epileptic seizure, referred to as an anoxic-epileptic seizure. These episodes are rare and less commonly reported, and may develop into status epilepticus [3]. Anoxic-epileptic seizures have also been reported in adults [4]. Here, we present a case of an adult patient with RAS, who experienced successful prevention of an episode using intranasal midazolam.

## Case presentation

Our patient is an 18-year-old Caucasian female who presented to outpatient neurology for the evaluation of “pain-induced seizures.” Her history included seizure-like episodes after needle stick procedures, such as blood draws and vaccinations. As a result, she had been given a medical exemption for vaccinations. She experienced episodes following other unpleasant stimuli including ear piercings and accidental cuts.

During an episode, the patient would become pale and lose consciousness, followed by generalized shaking movements and urinary incontinence. These episodes started at age 12 and were relatively infrequent. Her mother estimated five prior episodes. She had no prior EEGs and had never tried any medications for the episodes, but she was now more interested in treatment as she was leaving for college. Her only medication was sertraline for anxiety.

**Figure 1:**
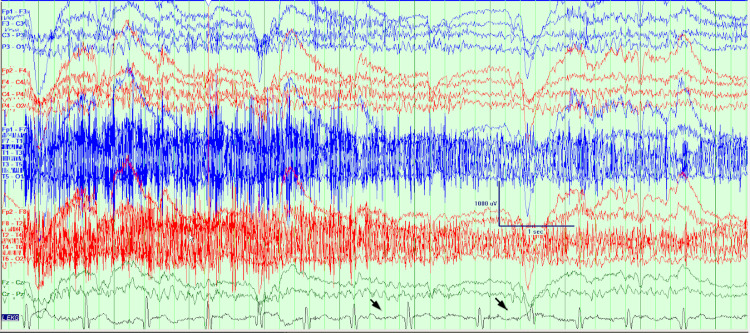
Baseline EEG with heart rate 72 beats per minute at the time of IV line insertion.

**Figure 2:**
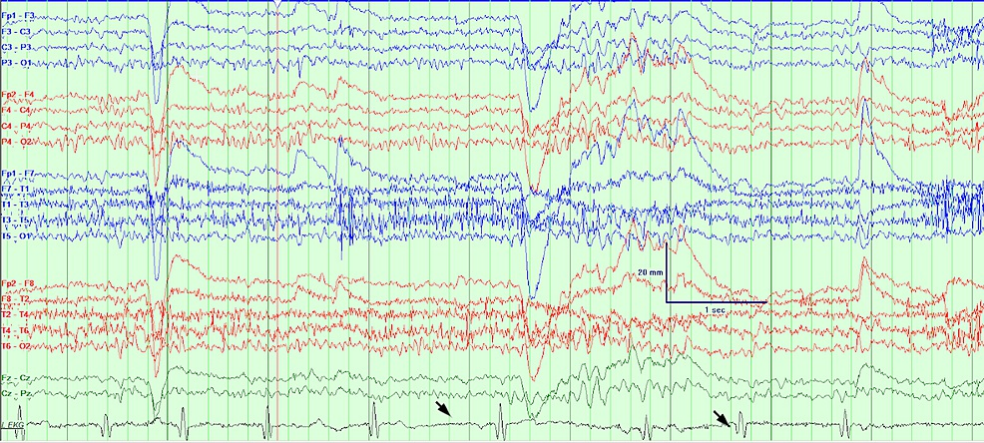
Ten seconds after the IV line was inserted, the patient became bradycardic.

**Figure 3:**
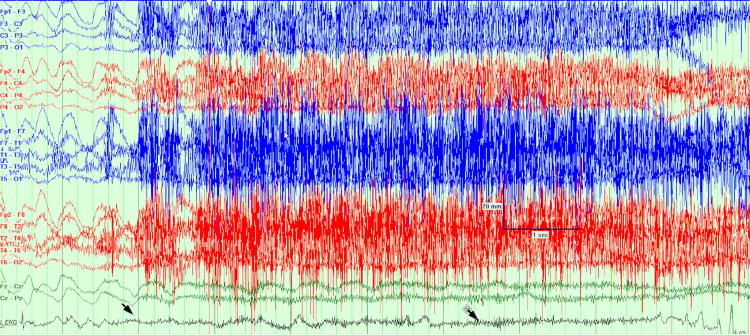
Asystole sets in, the patient is symptomatic and reports dizziness, speech mumbling noted, oxygen saturation drops.

**Figure 4:**
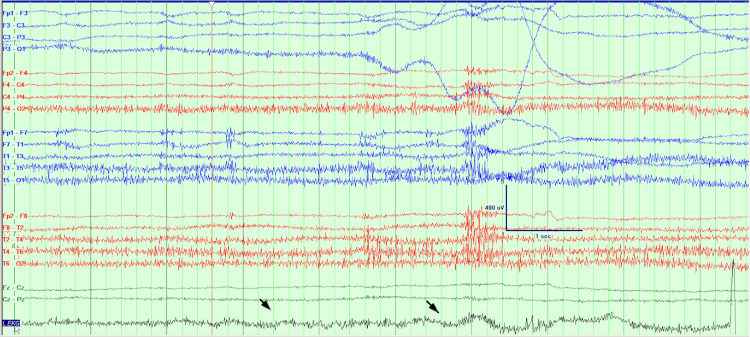
Asystole lasted 30 seconds.

**Figure 5:**
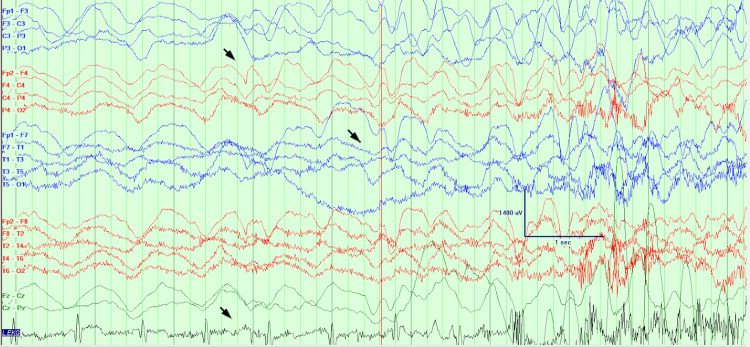
After 30 seconds of asystole, the heart rate slowly recovered. The patient had generalized clonic movements, EEG showed bilateral high amplitude rhythmic delta activity.

The patient was admitted to the epilepsy monitoring unit for further evaluation. MRI of the brain on admission was unremarkable and non-lesional for seizures. On the first day, after being pricked for an IV placement, she had a witnessed episode. Baseline monitoring is seen in Figure 1. Ten seconds after the attempted IV, her heart rate slowed (Figure 2). Approximately 30 seconds after the IV, she had 30 seconds of asystole (Figures 3, 4). She became pale and diaphoretic, followed by tonic posturing and loss of consciousness. Ninety seconds after the prick, she displayed clonic jerking lasting 10 seconds (Figure 5), along with urinary incontinence.

On the second day of admission, there was a second attempt at IV placement, with the patient connected to a 12-lead EKG. She had a second characteristic episode. EEG in both cases showed sudden onset bilateral delta slowing. Baseline EEG was otherwise normal. The patient was seen by cardiology and electrophysiology, and she was diagnosed with vagally mediated sinus arrest. A pacemaker was not recommended because the episodes were infrequent. The patient had no known family history of seizures or similar episodes, and interestingly her father did have a pacemaker for AV block.

A few days later, a trial was done with the patient receiving 5 mg intranasal midazolam one minute prior to an IV stick. She was able to remain alert and asymptomatic after this stick, with no syncope or seizure episode occurring. She was advised to use the medication prior to any blood draws or other known triggers to help prevent future episodes. Additionally, she was advised to use the medication if an accidental unpleasant stimulus occurred.

## Discussion

RAS occur when a stimulus, such as pain or fright, leads to a vagally mediated cardiac asystole, resulting in cerebral anoxia [5]. The asystole typically lasts under 15 seconds [5]. Convulsions then occur due to cerebral anoxia. This differs from epilepsy, where seizures occur due to abnormal neuronal discharges. EEG during asystole shows bilateral slow waves, which with continued asystole develops into bilateral attenuation [2]. Once the heartbeat returns, EEG again shows bilateral slow waves. It is during this transition time that true epileptic seizures can be triggered [2]. In our patient, painful stimuli such as needle sticks, led to 20-30 seconds of asystole, resulting in a syncopal episode with tonic posturing and clonic movements. This represents RAS. Though our patient has epileptic components of clonic movements, loss of consciousness, tongue biting, and urinary incontinence, this is more likely RAS than epileptic seizures. The patient was monitored for over 72 hours, with no interictal epileptiform discharges. Additionally, there was no dynamic evolution to electrographic or clinical seizures except the above-mentioned needle stick-induced events. This s­uggests that the patient did not have any underlying seizure diathesis or predisposition.

In a child with typical RAS episodes, the first part of treatment involves reassurance that brain damage is not occurring and education that the child will likely outgrow the episodes [1]. For patients with frequent episodes, medical management may include fludrocortisone or beta-blockers, although these treatments may work through a placebo effect [1]. Atropine is generally not used due to side effects [1]. Anti-epileptics should not be used in RAS, although their use in anoxic-epileptic seizures requires further research. In an observational study, anti-epileptic medications prevented the epileptic component, but not the syncopal component, in five out of seven cases [3]. Anti-epileptics were not recommended for our patient given her infrequent episodes which were only induced by pain. In severe cases of RAS, pacemakers may be used in children [1]. In adults, the ISSUE-3 trial found that dual-chamber pacemakers were an effective treatment in patients over 40 years of age with neurally mediated syncope with asystole [6]. Given that our patient was relatively young with infrequent episodes, a pacemaker was not recommended.

Our patient is 18 years old, and while there are reported cases of RAS in adults [7], it is much less common. Anxiety seemed to play a significant role in our patient’s vasovagal reaction. She was treated with intranasal midazolam to target her anxiety, which successfully prevented an episode. Midazolam can also be used as a rescue medication for patients with anoxic-epileptic seizures [5]. The patient was also on sertraline for anxiety, and she may benefit from cognitive-behavioral therapy to help target her reactions to unpleasant stimuli. This case demonstrates the importance of distinguishing RAS from epilepsy. It also demonstrates the ability to prevent anoxic seizures by targeting anxiety in select patients.

## Conclusions

RAS are rare and there is no consensus on treatment, this case is reported to support the literature for treatment of anoxic seizures. Further, this case is reported due to the rare finding of RAS in an adult patient, with full EEG recordings of an event. Our patient had a successful trial with intranasal midazolam, showing that benzodiazepines may be helpful for the prevention of episodes in similar patients. Prevention of episodes can significantly increase patient quality of life. Further research is needed on the treatment of RAS, as well as the role of anxiety medications in these patients.
